# CircIMPACT: An R Package to Explore Circular RNA Impact on Gene Expression and Pathways

**DOI:** 10.3390/genes12071044

**Published:** 2021-07-06

**Authors:** Alessia Buratin, Enrico Gaffo, Anna Dal Molin, Stefania Bortoluzzi

**Affiliations:** 1 Department of Biology, University of Padova, 35131 Padova, Italy; alessia.buratin.phd@gmail.com; 2 Department of Molecular Medicine, University of Padova, 35131 Padova, Italy; enrico.gaffo@unipd.it (E.G.); anna.dalmolin@unipd.it (A.D.M.); 3 Interdepartmental Research Center for Innovative Biotechnologies (CRIBI), University of Padova, 35131 Padova, Italy

**Keywords:** circular RNA, gene expression, regulatory axes, pathways

## Abstract

Circular RNAs (circRNAs) are transcripts generated by back-splicing. CircRNAs might regulate cellular processes by different mechanisms, including interaction with miRNAs and RNA-binding proteins. CircRNAs are pleiotropic molecules whose dysregulation has been linked to human diseases and can drive cancer by impacting gene expression and signaling pathways. The detection of circRNAs aberrantly expressed in disease conditions calls for the investigation of their functions. Here, we propose CircIMPACT, a bioinformatics tool for the integrative analysis of circRNA and gene expression data to facilitate the identification and visualization of the genes whose expression varies according to circRNA expression changes. This tool can highlight regulatory axes potentially governed by circRNAs, which can be prioritized for further experimental study. The usefulness of CircIMPACT is exemplified by a case study analysis of bladder cancer RNA-seq data. The link between circHIPK3 and heparanase (HPSE) expression, due to the circHIPK3-miR558-HPSE regulatory axis previously determined by experimental studies on cell lines, was successfully detected. CircIMPACT is freely available at GitHub.

## 1. Introduction

Circular RNAs (circRNAs) are a class of abundant and stable RNAs that result from the ligation of a downstream splice donor to an upstream splice acceptor [1]. The progressive discovery of circRNA functions, involvement in biological processes, and oncogenic potential made them attractive molecules for both fundamental and cancer research [2].

**Figure 1:**
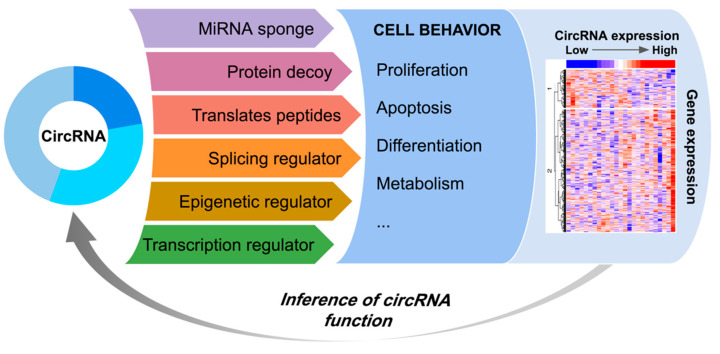
CircRNAs regulate cell behavior with different mechanisms. The integrated analysis of circRNA and linear gene expression profiles can predict circRNA functionsby identifying the biological processes and pathways impacted by circRNA expression variation.

CircRNAs regulate cellular processes by acting with different mechanisms (Figure 1), mostly involving sequence-specific binding with other nucleic acids or proteins. Of note, one prominent mechanism whereby circRNAs are believed to function is by sponging miRNA, thus regulating the expression of miRNA-target genes, working as competitive endogenous RNAs (ceRNAs) [3]. Aberrant DNA methylation and histone modifications can be controlled by circRNAs that regulate key epigenetic “writers” like DNMT1 [4] and EZH2 [5] methyltransferases. Regulatory functions on gene transcription were also described for circRNAs [6]. Other circRNAs modulate the activity of RNA-binding proteins (RBPs), a large class of molecules involved in a multitude of processes, including the control of cell cycle progression [7] and splicing [8], among others. In addition, since most circRNAs originate from the circularization of coding gene exons, circRNA biogenesis can compete with linear RNA splicing [8]. Beyond exerting functions typical of long non-coding RNAs, circRNAs can be translated into peptides [9,10].

Evidence on circRNA functions added another level of complexity in the network of diverse players in regulating cellular processes, impacting normal and pathogenic or malignant phenotypes. Transcriptomics data has been proven useful to unearth the effects of protein and RNA dysfunctions, and their contribution to the pathogenesis and global deregulation of gene expression in cancer. This type of analysis falls in the field of expression data reverse engineering, which has been extensively used to infer regulatory networks involving transcriptional regulators [11], miRNAs [12], and combinations thereof [13,14]. Starting from the hypothesis that circRNAs affect gene expression, then gene expression profiles could be used to infer circRNA biological functions (Figure 1).

CircRNAs can be detected and quantified using RNA-seq [15] and appropriate software tools [16,17]. Typically, studies aiming at identifying circRNA roles in disease and cancer face two consecutive challenges. The first is to efficiently prioritize the circRNAs that discriminate between normal and malignant cells or that have the potential to define new and possibly relevant disease subtypes. Subsequently, the involvement of circRNAs in specific pathological mechanisms or biological processes should be identified. These challenges can be tackled by circRNA screening, for instance, by massive circRNA silencing or overexpression studies, and with experimental investigation of the mechanisms involved, once the circRNAs whose expression impacts significant cell features have been identified.

Computational predictions were extensively used to identify circRNA correlation with diseases [18] and infer circRNA functions. Most of the efforts were put toward predicting the miRNA-sponging activity of circRNAs and inferring circRNA-miRNA-gene regulatory axes. Among circRNA-dedicated databases, CircInteractome [19], CircAtlas [20], and CSCD [21] provide information about miRNAs potentially sponged by circRNAs. However, none of them allows the analysis of new expression data.

The ceRNA function was the first described for circRNAs [1,22] and has important implications in cancer. We and other groups used custom integrative analysis of circRNA, miRNA, and gene expression data, with systems biology methods, to infer circRNA regulatory networks [23,24,25,26]. Besides, the Cerina tool for predicting biological functions of circRNAs based on the ceRNA model was recently made available [27]. However, the ceRNA function may not apply to all circRNAs [28,29]: systematic analyses of circRNA sequences showed that only a minority present multiple binding sites for specific miRNAs [30], and, in general, circRNAs are not bound to miRNA-loaded Argonaute proteins [31]. As described above, robust evidence disclosed that circRNA functions can be exerted by several different mechanisms (Figure 1).

Recent studies provided interesting hints on circRNA functions comparing the gene expression between sample groups defined by diverging expression of one specific circRNA. For example, a high circFBXW7 expression in acute myeloid leukemia (AML) patients was linked to gene expression profiles enriched in genes encoding epigenetic regulators and transcription factors governing leukocyte activation. Conversely, a low circFBXW7 abundance was linked to cell stemness [32]. Similarly, circBCL11B expression in AML patients has been associated with a T-cell–like gene expression signature [33]. These observations prompted the effectiveness and usefulness of predicting the circRNA impact on gene expression and cell behavior, independently of specific or known circRNA function mechanisms.

To our knowledge, currently available bioinformatics tools do not support the integrated analysis of circRNA and gene expression profiles to prioritize the most promising candidate circRNAs whose expression changes can have an impact in biological processes, pathways, and ultimately cell phenotypes. Therefore, we have developed CircIMPACT (https://github.com/AFBuratin/circIMPACT), an R package. It offers a comprehensive pipeline that integrates different approaches to select circRNAs discriminating sample groups by unsupervised analysis, allows integrative analysis of circRNAs and gene expression data to define gene differentially expressed between and most discriminating samples groups defined by circRNA expression changes. Finally, functional enrichment analysis helps the user to identify the biological processes and signaling pathways impacted by each circRNAs.

## 2. Materials and Methods

### 2.1. Input Data and Format

The program’s workflow for circRNA impact analysis begins with the loading of (i) back-splice junction read count, (ii) gene expression quantifications in the same samples, and (iii) sample clinical data. CircIMPACT does not require input files generated with specific circRNA/gene expression quantification methods. The user-owned files are only required to be properly formatted as follows: each row of the expression matrices must contain circRNA or gene expression values, with one column per sample; the first column must contain the circRNA or the gene identifiers, and the first row the sample names. The sample clinical data must be declared in a table format with samples in rows and data sources in columns. The CircIMPACT input file formats resemble the expression matrix and metadata input formats required to build a SummarizedExperiment object [34]. In this work, we leveraged the CirComPara2 software to obtain expression input files from RNAseq data analysis. However, other or custom computational pipelines can be used for this purpose.

The circRNA and gene expression matrices are normalized by raw library size scaling using the *sizeFactors* function from the R package *DESeq2* [35] for visualization. Normalized counts are computed using the function *counts* from *DESeq2*.

### 2.2. CircRNA Selection and Sample Grouping

**Figure 2:**
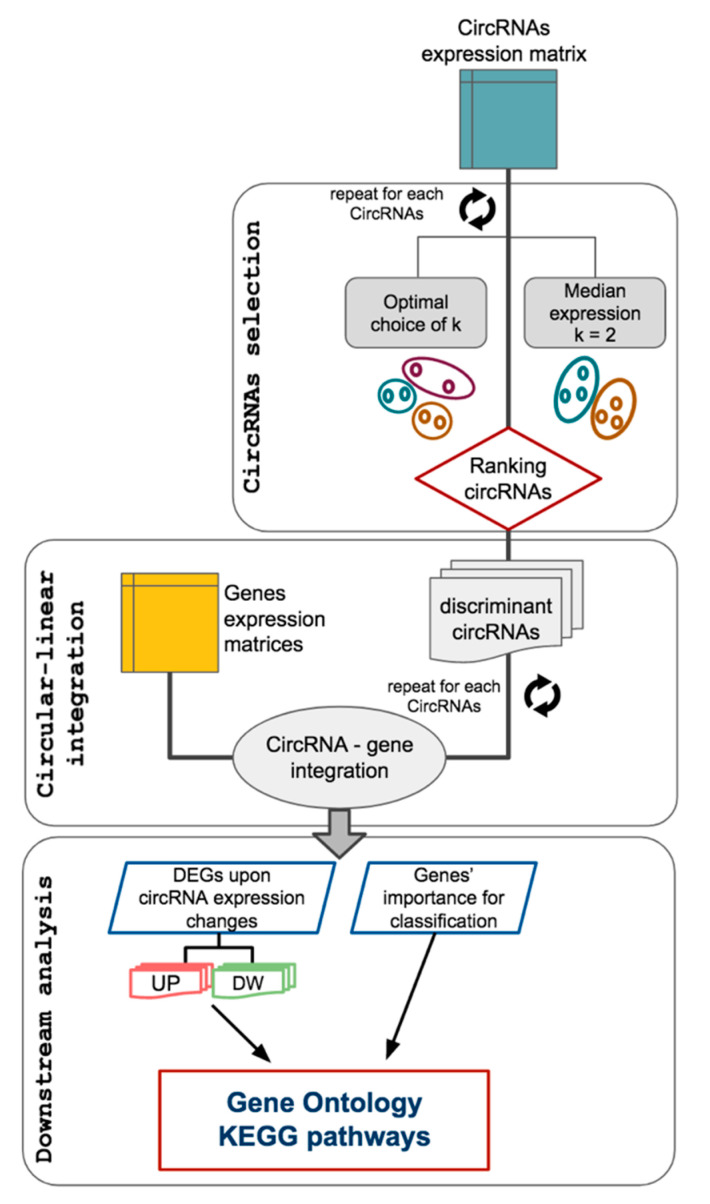
The CircIMPACT workflow.

The first step of the CircIMPACT workflow (Figure 2) is to compute sample groups according to the expression pattern of each circRNA. This process is independent of the sample annotation provided by the user, and, in principle, each circRNA may determine a different grouping of the samples. Then, for each circRNA expression-defined sample group, the circRNAs are tested to obtain significance estimates of the expression change between the groups. After correction for multiple tests, the circRNAs with significant expression variation are selected.

The function *marker.selection* (Table 1) fulfills this task. The user can choose between two approaches to defining the circRNA expression-guided sample grouping: the default one splits the samples into two groups according to the circRNA median expression; the second strategy is based on clustering. If the clustering method is chosen, the user can customize the function behavior through several options: distance measure and agglomeration method of the clustering, a fixed number of final clusters (i.e., sample groups), or optimal cluster number data-driven computation. The parameters available for these options derive from the *dist* and *hclust* functions of the R package *stats* and the *NbClust* R package [36]. Default settings consider Euclidean distance, k-means agglomeration, and automatic determination of the optimal number of clusters using the *silhouette* index parameter for the NbClust function. We recommend the use of the default method with small sample size, when only two groups are expected and for a first exploratory analysis. Sample clustering can be useful with large datasets and to discover sample groups, beyond annotation.

In the median expression threshold approach, the circRNAs undergo pairwise contrast differential expression tests performed using the *nbinomWaldTest* function from the *DESeq2* package. Only circRNAs with an adjusted *p*-value ≤ 0.05 and absolute log_2_ fold-change ≥ 1 are selected for further analysis. Whereas, with the clustering approach, the circRNAs are selected according to one-way ANOVA test *p*-values ≤ 0.05 calculated using the function *anova* from the R *stats* package.

CircRNA passing these filters compose the list of the discriminant circRNAs for the corresponding sample grouping.

### 2.3. Dimensionality Reduction and CircRNA Ranking

Principal component analysis (PCA) is used to perform dimensionality reduction of the appropriately standardized circRNA expression matrix to cluster samples and calculate the goodness of sample separation, according to the circRNA expression. This analysis takes as input back-splicing quantification tables, with the samples of interest as rows and the selected circRNAs as columns. PCA is implemented through the singular value decomposition algorithm provided by the *prcomp* function from R package *stats*.

The total contribution of each variable (circRNA) towards data variance along with selected principal components, based on the implementation of *fviz_contrib* from R package *factoextra*, is used to rank the circRNAs and narrow down the list of circRNAs kept for further investigation. By default, 25 circRNAs most-discriminating among groups are selected; this number is nevertheless customizable by the user.

### 2.4. Differential Gene Expression

The differential gene expression analysis is implemented in the *gene.expression* function (Table 1) and is based on fitting a negative binomial model through the *DESeq* function of the *DESEq2* package for the sample groups defined with the *marker.selection* function. *p*-Values are corrected for multiple tests with the Benjamini–Hochberg procedure. The non-normalized gene count matrix is required for this analysis.

A result table is provided including, for each gene significantly associated with the circRNA expression pattern, its average expression across samples, the logarithm fold change and the corresponding standard error, the statistic of the DEseq test, the *p*-value, and the adjusted *p*-value. This table can be saved as a .csv file.

### 2.5. Classification Analysis

The CircIMPACT *gene.class* function selects the best putative predictors (genes) for the classification of samples in the groups defined by the expression changes of a given circRNA (see Table 1). It is structured in three main processes: normalization, selection, and classification. Normalization includes basic preprocessing of raw gene counts and data transformation. A ‘Feature Selection’ procedure to extract a small subset of informative genes from the original data is implemented based on the backward variable elimination (partial least squares) to remove the less informative variables with respect to the response variable, including those redundant with the selected variables highly correlated with the sample groups [37]. Finally, the random forest classification model is used to classify the sample groups according to gene expression and allow the reordering of genes to rank their importance. CircRNA host genes are not treated in a specific way, but are analyzed in the same way as all the other genes. The *rfe* and *varImp* functions from the R package *caret* are used for classification and variable importance reordering, respectively. The R package *randomForestExplainer* is used to visualize the results of the random forest model.

### 2.6. Functional Enrichments

CircIMPACT supports the gene set enrichment analysis (GSEA). In particular, the *gseGO* and *gseKEGG* functions from the R package *enrichplot* are used for functional enrichment analysis of genes associated to a given circRNA, whereas the *dotplot* and *upsetplot* functions are used to help to visualize and interpret the enrichment results.

### 2.7. Sample Analysis

Bladder cancer RNA-seq data (GSE97239) [38] were downloaded from Gene Expression Omnibus database (https://www.ncbi.nlm.nih.gov/geo/).

CircRNA and gene expression quantification matrices used as input for CircIMPACT were computed with CirComPara2 v0.1.2.1 [16] applied with default parameters. Default CircIMPACT settings were used unless otherwise stated, including median threshold, for group separation, according to the experimental design and size of the sample analysis data. CircRNAs and genes supported by at least 5 and 20 reads, respectively, in half of the samples were kept. In addition to CircIMPACT output files and figures, density and volcano plots were generated using customized R functions to visualize results.

### 2.8. Software and Versions

The CircIMPACT tool is developed based on R [39] (R version 3.6.3 or later are recommended), which also depends on several R packages (knitr [40], rmarkdown [41], data.table [42], dplyr [43], tydyverse [44], Rtsne [45], kableExtra [46], sparkline, magrittr [47], caret [48]). Additional R packages (ggplot2 [49], ComplexHeatmap [49,50], circlize [51]) were used to produce the figures in this paper.

## 3. Results

### 3.1. CircIMPACT Workflow

CircIMPACT has been implemented as an R package that integrates circRNA expression with gene expression to allow researchers to identify the most condition-discriminant circRNAs and to predict their impact on gene expression, as a proxy for cell behavior (Figure 1). To this purpose, CircIMPACT offers a comprehensive pipeline to perform dimensionality reduction on circRNA expression profiles, differential circRNA and gene expression analysis, and functional enrichments. The tool can be applied in different contexts, ranging from the simple contrast between normal and tumor samples to more complex and heterogeneous disease sample groups.

CircIMPACT needs three input sources: (i) a circRNA expression matrix, (ii) a gene expression matrix, and (iii) the sample metadata containing, for instance, sample biological or clinical information (Figure 2). The analysis workflow is summarized in Figure 2. Data are filtered based on user-provided parameters to eliminate weakly expressed circRNAs and genes. Normalized expression values are computed for later visualization. At first, circRNA expression data are analyzed to pick circRNAs able to discriminate samples into two or more groups for subsequent analysis. Next, gene expression data are taken into account and integrated with circRNA variations across samples. For each of the circRNA selected, differential expression and classification analysis are performed over gene expression data to retrieve genes dysregulated according to circRNA expression changes across samples. Finally, gene enrichment tests show the pathways and functions correlated with each circRNA and potentially controlled by its activity through different mechanisms.

### 3.2. Case Study: An Exploration of Gene Expression and Pathways Impacted by circRNA in Bladder Cancer

We applied CircIMPACT to a real dataset, to illustrate the package functions and output, as well as to demonstrate its discovery power. We selected RNA-seq data originally produced by a bladder cancer study that profiled tumor tissue of three patients and the matched normal counterpart, detecting massive circRNA dysregulation in the tumor [38]. Bladder cancer is the most commonly occurring tumor of the urinary system and the ninth most frequently diagnosed cancer in the world [52]. In the same study, the role of circHIPK3 downregulation has been subsequently demonstrated through a series of functional experiments in vitro. By circHIPK3 enforced expression, the authors showed that this circRNA suppresses invasion, metastasis, and angiogenesis of bladder cancer cells, by repressing heparanase (HPSE) expression via sponging miR-558. Of note, unsupervised CircIMPACT analysis of the original patient RNA-seq data successfully detected HPSE expression dependence from circHIPK3 level, unearthing the unconventional circHIPK3-miR558-HPSE regulatory axis previously described by experimental studies [38]. Notably, most miRNAs have a suppressive function on their target genes, acting as negative post-transcriptional regulators. In “classic” circRNA/miRNA/gene regulatory axes, concatenation of two repressive regulatory relations determines a positive correlation between the expression profile of the circRNA and the gene targeted by the miRNA decoyed by the circRNA [26,27,28,29]. Differently, miR-558 was proven to act with a different mechanism, to positively regulate HPSE transcription, sustaining mRNA production [30]. Thus, in the normal tissue, circHIPK3 efficiently sponges miR-558 leading to HPSE repression. Reduced circHIPK3 expression in bladder cancer derepresses the enzyme whose activity unleashes cancer cell metastatic potential. Consequently, the circHIPK3-miR-558-HPSE axis determines a negative correlation between circHIPK3 and HPSE expression.

**Figure 3:**
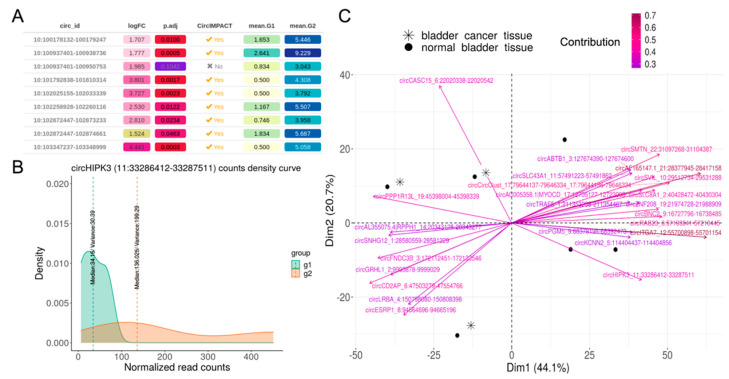
CircRNA analysis of bladder cancer RNA-seq data. (**A**) Fragment of the table produced by the *circ.marker* function, indicating circRNAs equally or differentially expressed among sample groups. (**B**) Density plot for circHIPK3 expression in the sample groups defined by circHIPK3 median expression (g1 corresponds to bladder cancer, g2 to control samples). (**C**) Principal component analysis (PCA) of the sample separation using the top 25 circRNAs mainly contributing to sample separation, which include circHIPK3.

CircComPara2 analysis of the bladder cancer dataset produced expression matrices of 50,513 circRNA and 51,151 genes. Analysis by CircIMPACT deemed 3479 circRNAs to be differentially expressed (DE) between the two groups defined by the circular median expression, which were saved in a table (Figure 3A).

In accordance with Li et al. [24], among the differentially expressed circRNAs, CircIMPACT identified circHIPK3, which is downregulated in the automatically defined group (g1), corresponding to bladder cancer samples (Figure 3B). Different circRNAs can separate sample groups in different ways, according to the experimental design, the dataset type and structure. In this case, the group separation according to all DE circRNAs taken together separates bladder cancer and normal tissue samples.

Afterwards, CircIMPACT selected circRNAs that contribute most to the sample separation in the two first principal components. Notably, the top 25 circRNAs identified (Figure 3C and Table 2) included circHIPK3. We verified that running CircIMPACT with the k-means option for sample separation, circHIPK3 remains in the top 25.

**Figure 4:**
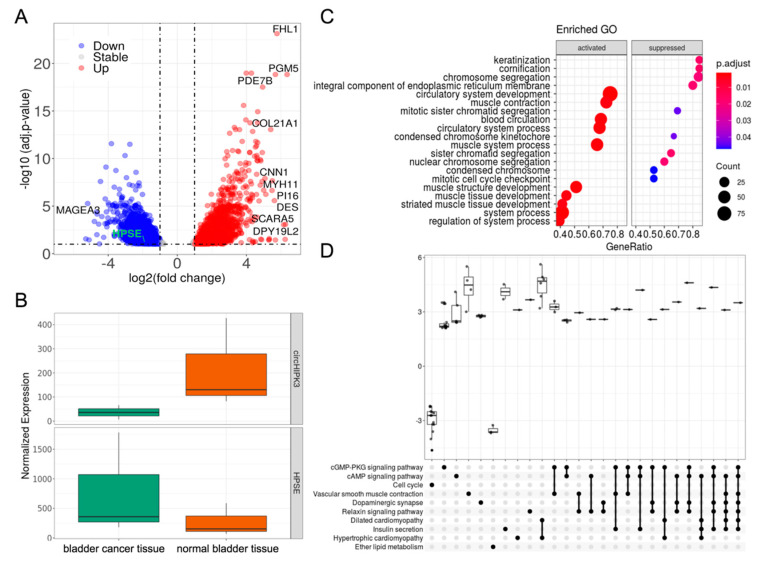
Putative impact of circHIPK3 in gene expression, gene ontology, and pathways. (**A**) Volcano plot of the differentially expressed genes in sample groups defined by circHIPK3 expression. (**B**) CircHIPK3 and the HPSE gene are respectively up- and downregulated in bladder cancer. (**C**) Top 10 activated and suppressed GO enriched in differentially expressed genes in the group comparisons (**D**) Pathways most enriched in deregulated genes.

Next, for each circRNA, the differentially expressed genes were identified in the corresponding sample groups. CircIMPACT revealed, in association with circHIPK3 expression changes, 1124 genes down- and 1236 genes upregulated in the sample group 2 characterized by the highest expression of circHIPK3 (Figure 4A). Of note, we found a significant downregulation of HPSE when circHIPK3 is upregulated (Figure 4B).

CircIMPACT allows performing functional enrichment tests. Running the analysis on DE genes associated with circHIPK3 variation, we identified 746 Gene Ontology (GO) categories significantly enriched in the DE genes associated with circHIPK3 (p.adj ≤ 0.05). The ten most enriched GO terms are shown in Figure 4C. The most enriched KEGG pathways include cell-cycle, with genes less expressed in the group with circKIPK3 upregulated, in other words, in normal tissue compared to bladder cancer. In line with their tumor suppressor role, cGMP-PKG [31] and cAMP [32] signaling pathways are enriched among genes more expressed in normal tissue (Figure 4D).

**Figure 5:**
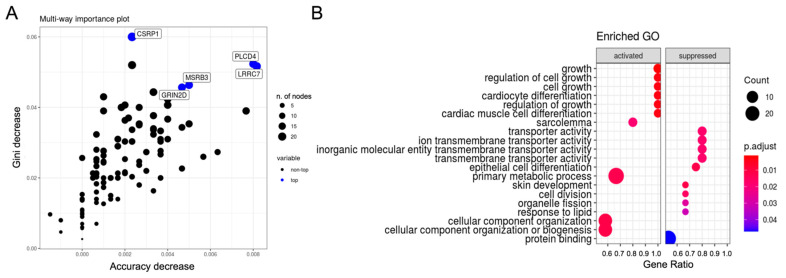
The most important genes in the classification analysis of sample groups, defined by circHIPK3 expression. (**A**) Variable importance plot of the most relevant genes used in the random forest model; (**B**) top 10 activated and suppressed enriched GO using most important genes in the group classification.

In addition, for each circRNA, CircIMPACT identified the subset of genes being the best putative predictors of sample classification in the two groups defined by the expression changes of the circRNA. The importance plot in Figure 5A shows the best predictors for circHIPK3 in our sample analysis. The corresponding GO functional enrichment analysis results are shown in Figure 5B.

## 4. Discussion

In this study, we developed a bioinformatic software package dedicated to integrated analysis of circRNA and gene expression to investigate the possible impact of circRNA in gene expression, biological functions, and pathway activation. CircIMPACT is a freely available R package incorporating several user-friendly features that can facilitate the prediction of circRNA functions and the identification of genes and pathways dysregulated according to circRNA expression changes. In this way, CircIMPACT has the potential to pinpoint specific regulatory axes potentially governed by circRNAs, highly pleiotropic molecules, and to prioritize some of them for further experimental study.

CircRNA functions are exerted mainly through mediators, such as miRNAs and RNA-binding proteins (RBPs) or encoded peptides. The activities mediated by miRNAs, although indirect, can significantly affect gene expression, in a way that is predictable: when a circRNA acts as a decoy for a miRNA, the circRNA upregulation desuppresses miRNA target genes, likely resulting in their upregulation. Thus, circRNA and gene expression changes can be used to investigate and also discover circRNA-miRNA-gene axes. With this type of regulatory axis, it should be noticed that the expression of miRNA can be unaffected by the circRNA differential expression. Since the miRNA is still present, but it is sequestered by the circRNA, only the expression of miRNA target genes is varied. This concept argues against an inclusion of the miRNA expression data in the analyses. Once a possible link between a circRNA and a gene is detected, as with CircIMPACT analysis, possibly involved miRNAs can be retrieved from databases, such as miRTarBase, a curated database of microRNA-target interactions.

Other functions of circRNAs can be more difficult to predict using gene expression data, such as those entailing circRNA-encoded peptides and interactions of circRNAs with RBPs. As an example, a circRNA can both facilitate (e.g., acting as scaffold for a molecular complex) and counteract (e.g., decoying the RBP) protein functions. However, if this protein’s functions, in some way, regulate cell behavior or gene expression, integrative analysis of circRNA and gene expression should provide a molecular readout of the circRNA expression changes and of the genes and biological processes influenced by the circRNA.

To demonstrate CircIMPACT functions and potential we conducted a sample analysis using bladder cancer and matched control tissue data. CircIMPACT analysis identified HPSE dysregulation according to circHIPK3 expression reduction in bladder cancer, in line with the non-canonical regulatory axis previously proven and linked to the disease [24]. In our sample analysis, the software was applied to a relatively small and simple dataset. This choice was based on the availability of ribodepleted RNA-seq data adequate to study both circRNA and gene expression and previously established functional evidence about the function of a specific circRNA that could be used for indirect method validation. In this specific case, using both the circRNA expression median as a threshold and the k-means clustering approach with the optimal number of clusters estimation, samples were clustered as in the annotation groups, i.e., separating cancer and normal tissue samples.

In addition to circHIPK3, the integrative analysis of publicly available bladder cancer RNA-seq data by CircIMPACT identified other circRNAs discriminating between sample groups, linking them to gene expression changes. These new hints indicated circRNAs that are worth study, to provide novel insights into bladder cancer. For instance, circZNF208 (19:21974728-21988909) and circFER1L4 (20:35595476-35595754), resulting in respectively down- and upregulation in bladder cancer, were previously detected by Li et al. [24] but not functionally investigated, whereas they were selected in the top 25 circRNAs by our analysis. Both circZNF208 and circFER1L4 are poorly characterized and might deserve further study.

It is important to note that, beyond the classic design entailing comparison of a disease condition with its normal counterpart, CircIMPACT can be applied to more complex datasets. The clustering, dimensionality reduction, and classification functions implemented in CircIMPACT will likely gain discovery power when used on large datasets of at least 30 samples. Plus, the unsupervised analysis of sizable patient cohorts has the potential to discover new disease subtypes defined by circRNA expression. In turn, a “footprint” of circRNA expression variation in gene expression profiles can be identified, indicating the pathways and functions as well as those putatively impacted by the identified discriminating circRNAs. In this discovery-driven analysis, the new groups defined by circRNA expression can be transversal to the groups defined by a priori sample annotation. They could secondarily be put into relation with biological features of the samples or with the patient clinical data, to further study the involved mechanisms and possible clinical correlations.

Currently, CircIMPACT supports analysis based on RNA-seq read counts. In our sample analysis, we used the same RNA-seq data to estimate both the circRNA and the gene expression matrices. In principle, circRNA and gene expression can be estimated with different methods in the same set of samples. For instance, qRT-PCR quantification of a circRNA panel in samples extracted from available patient biobanks can be integrated with genome-wide gene expression data previously obtained with microarray analysis of the same cases. In the near future, the incorporation of functions adequate for managing different types of expression quantification data will enrich the package and broaden its scope. Certainly, it is of utmost importance to promote circRNA analysis of biological and clinical samples as a crucial complement to more conventional research focused on gene expression.

## 5. Conclusions

In conclusion, CircIMPACT R package allows integrated analysis of circRNA and gene expression to investigate the functional impact of circRNA expression variation and to explore possible sample groups defined by circRNA expression level, with the potential to discover new disease subtypes. Certainly, it is of utmost importance to promote circRNA analysis of biological and clinical samples as a crucial complement to more conventional research focused on gene expression.

## Figures and Tables

**Table 1:** The functions implemented in CircIMPACT.

Function	Description
*marker.selection*	Defines the discriminant circRNAs and sample grouping according to circRNA expression patterns
*gene.expression*	Performs differential gene expression tests between the sample groups defined by circRNA expression
*gene.class*	Normalization, feature selection, and classification of sample groups defined by circRNA expression using gene expression

**Table 2:** Top 25 circRNAs discriminating better among sample groups. For each circRNA, identified by the circRNA host-gene name and the back-splice junction genomic coordinates (BSJ), the average expression in the two sample groups (g1 and g2) are indicated along with the circRNA fold change in base-2 logarithm (Log_2_FC) and the adjusted *p*-value (p.adj) of differential expression.

CircRNA Host Gene ^1^	Back-Splice Coordinates	Log_2_FC	p.adj	Mean g1	Mean g2
SVIL	10:29512734-29531288	7.3	0.0000	0.5	47.5
AMY2B	1:103565434-103575540	4.5	0.0000	2.3	27.1
HIPK3	11:33286412-33287511	2.5	0.0979	37.0	213.8
SLC43A1	11:57491223-57491862	3.2	0.0000	6.4	43.0
TRAF5	1:211353238-211354467	3.1	0.0000	5.1	44.3
ITGA7	12:55700898-55701154	5.3	0.0007	22.5	903.0
SNHG12	1:28580559-28581229	6.2	0.0000	1.0	25.9
RPPH1	14:20343123-20343272	3.2	0.0000	41.4	359.3
RPPH1	14:20343123-20343277	5.1	0.0008	4.3	122.8
RPPH1	14:20343128-20343277	3.3	0.0000	39.0	373.9
MYOCD	17:12705127-12723008	6.8	0.0000	0.5	32.8
CircClust	17:79644136-79646334	5.4	0.0003	2.2	83.4
ZNF208	19:21974728-21988909	3.0	0.0000	52.7	419.3
PPP1R13L	19:45398004-45398339	4.2	0.0215	3.8	55.9
FER1L4	20:35595476-35595754	3.2	0.0097	4.5	36.0
AF165147.1	21:28377945-28417158	6.4	0.0000	1.8	145.7
SMTN	22:31097268-31104387	6.3	0.0001	1.2	71.9
SLC8A1	2:40428472-40430304	3.3	0.0007	7.0	59.2
GRHL1	2:9995878-9999029	4.5	0.0000	2.4	30.3
ABTB1	3:127674390-127674600	3.8	0.0000	3.7	39.3
FNDC3B	3:172112451-172133546	3.5	0.0000	8.3	86.1
KCNN2	5:114404437-114404856	4.7	0.0008	2.2	56.6
CD2AP	6:47503279-47554766	4.5	0.0153	3.1	62.3
RAB23	6:57193841-57210445	5.4	0.0001	1.5	53.8
PGM5	9:68378198-68392473	4.5	0.0009	1.5	30.5

^1^ CircRNA annotation was based on the Ensembl GRCh38 human genome and annotation v93. Genomic regions without annotated genes but expressing one circRNA or more circRNAs (overlapping or not more than 5000 nt apart) defined new loci, called “CircClust”.

## Data Availability

CircIMPACT is an open-source R package publicly available in Github at https://github.com/AFBuratin/circIMPACT along with command-line interface based on the presented case study.
